# The Project P.A.T.H.S. in Hong Kong—Lessons Learned and Implications for Positive Youth Development Programs

**DOI:** 10.1100/2012/687536

**Published:** 2012-04-01

**Authors:** Daniel T. L. Shek, Rachel C. F. Sun

**Affiliations:** ^1^Department of Applied Social Sciences, The Hong Kong Polytechnic University, Hong Kong; ^2^Public Policy Research Institute, The Hong Kong Polytechnic University, Hong Kong; ^3^Department of Sociology, East China Normal University, Shanghai 200241, China; ^4^Kiang Wu Nursing College of Macau, Macau; ^5^Division of Adolescent Medicine, Department of Pediatrics, Kentucky Children's Hospital, University of Kentucky College of Medicine, Lexington, KY 40506-9983, USA; ^6^Faculty of Education, The University of Hong Kong, Hong Kong

## Abstract

The Project P.A.T.H.S. is a positive youth development program which attempts to promote holistic development of junior secondary schools in Hong Kong. It is ground breaking in terms of the number of schools participating in the project and financial resources injected into the project. Based on the experiences gained from the implementation of the project and evaluation data collected from 2005 to 2011, several issues pertinent to the development of positive youth development programs in the Chinese culture are discussed. These issues include complexity of program development, importance of training, identification of factors governing program implementation, need for evaluation, and promotion of sustainability of the program in the long run.

## 1. Introduction

With the intensification of adolescent issues, increasing effort has been paid to the development of adolescent prevention and positive youth development programs in the global context. With specific reference to Hong Kong, there are also growing adolescent problems such as substance abuse, bullying, and suicide [1]. In response to such phenomena, a positive youth development program entitled Positive Adolescent Training through Holistic Social Programmes (Project P.A.T.H.S.) was initiated by The Hong Kong Jockey Club Charities Trust in 2004 to promote the holistic development of Chinese adolescents, with an earmarked grant of HK$400 million. Because of the positive evaluation findings of the initial phase, an extension phase was further implemented from 2009 to 2012, with an additional earmarked grant of HK$350 million [2].

The basic arguments for having positive youth development program are that many adolescent developmental problems have common origins and that adolescents have potentials and they are resources to be developed. Furthermore, one attractive feature of positive youth development programs is that instead of having many prevention programs for adolescent problems, one single positive youth development program may be enough to tackle different types of adolescent developmental tasks. As the evaluation papers in this special issue and elsewhere clearly demonstrate, the Project P.A.T.H.S. is effective in promoting the holistic development and reducing risk behavior of adolescents in Hong Kong.

Shek and Yu [3] pointed out that there is severe lack of adolescent prevention and positive youth development programs in different Chinese communities. As such, the Project P.A.T.H.S. can be regarded as pioneering and ground breaking in different Chinese communities, both in terms of the huge financial resources earmarked for the project, the large number of schools participating in the project, and the high degree of collaboration among the government, academics, welfare sector, and the education sector. Furthermore, valuable experiences regarding the development and implementation of positive youth development programs in Chinese communities are also accumulated. Obviously, with the conclusion of the first phase of the Project P.A.T.H.S., it is important to ask what lessons have been learned which can serve as useful pointers for future work in positive youth development in the Chinese culture.

Generally speaking, there are several steps in the development of positive youth development programs. According to Weissberg et al. [4] there are five stages intrinsic to the conceptual framework for conceptualizing, designing, implementing, and disseminating school-based social competence promotion programs. The first stage is* conceptualization* which involves the identification of personal resources (such as skills, knowledge, and beliefs) and environmental supports and their combinations that promote social competence in specified domains and/or prevent mental health problems based on theories, research findings, and intervention experiences. The second stage is *design* in which developmentally and culturally appropriate programs with application of effective classroom teaching principles are designed. The third stage is *implementation* where the program is adapted to the ecology of the school setting and that mechanisms are used to monitor the program integrity. The fourth stage is *evaluation* where the researchers examine whether the program is implemented effectively and the program improves the personal resources of the students. The fifth stage is *maintenance and dissemination* where issues of sustainability of the program and dissemination are considered. In the present paper, discussion and personal reflections with reference to these steps would be carried out.

## 2. Lessons Learned from the Project

### 2.1. Lesson  1: Program Development Is a Complex Matter Which Should Not Be Treated Lightly

In the education and welfare sectors in Hong Kong, there are many “home-baked” youth development programs as if such programs can be easily designed. One common example is that there are life skills or life education programs in many schools which are simply based on arm-chair theorizing and workers' professional judgment. While workers' judgment must be treasured, there is no guarantee that the related knowledge is beneficial to the students in the long run. Therefore, the intervention program to be developed must be guided by research findings and an evidence-based approach is called for. In the Project P.A.T.H.S., the following assertions are maintained in the conceptual model underling the proposed program.

**Assertion 1**

Based on ecological models, adolescent developmental outcomes are determined by personal factors (such as psychosocial competencies and beliefs) and environmental factors in different systems in the school, family, peers, community, and cultural contexts (i.e., ecological assertion).

**Assertion 2**

Based on the ecological perspective, changes in adolescents and their developmental contexts would be necessary to promote positive youth development (i.e., change assertion).

**Assertion 3**

Based on models on holistic health, existential theories, and transpersonal literature, it is important to consider adolescent development in the physical, psychological, social, and spiritual domains and to examine how such factors are related to adolescent development (i.e., holistic assertion).

**Assertion 4**

Based on life span developmental theories, there are different developmental assets to be developed by an adolescent (i.e., developmental assets assertion). In connection with this, adolescents have to learn different skills in their developmental trajectory.

**Assertion 5**

Risk factors in different ecological systems impair adolescent development (i.e., risk factors assertion).

**Assertion 6**

Protective factors in different ecological systems help an adolescent to cope with the negative impact generated by the risk factors (i.e., protective factors assertion).

**Assertion 7**

Besides focusing on the reduction of youth problem behavior (i.e., prevention science approach), focus should also be expanded to developmental potentials of adolescents (i.e., positive youth development assertion).

**Assertion 8**

Based on the literature on positive youth development, there are constructs that promote positive youth development (i.e., positive youth development constructs assertion).

**Assertion 9**

An integration of the positive youth development approach and the prevention science approach would be desirable (i.e., integration assertion).

**Assertion 10**

Existing theories, research findings, and available successful programs will be used to guide the conceptualization, design, implementation, and evaluation of the project (i.e., evidence-based assertion).

In future, several questions should be carefully considered as far as program development is concerned. Is positive youth development program a panacea to different kinds of adolescent developmental problems? Are different positive youth development constructs equally effective in solving all adolescent developmental problems? What are the theoretical and empirical bases of the developed program? Is the proposed program based on the best available evidence in the field? Ideally speaking, a well-conceived program should specify the theoretical mechanisms that are conducive to the program effects (e.g., modeling, experiential learning, and cognitive restructuring) and underlying the teaching and learning processes, cultural factors that may affect the program design and outcome, and the relationship between the program to be developed and the best available evidence. In addition, multidisciplinary collaboration among different professionals in designing and implementing the program is regarded as the ideal scenario.

### 2.2. Lesson  2: Training Programs for Potential Program Implementers Are Important

A review of the literature shows that training plays an important role in the smooth implementation and positive outcomes of positive youth development programs. As such, great effort was spent on training programs in the project, with 20 hours of training required for the potential program implementers at each grade. The following principles were adopted in the design of training programs.

**Design of Training Program Based on Training Theories/Models (Principle 1)**

It is important for training program designers to be conscious of the theoretical and conceptual bases of the training program.

**Acquisition of Knowledge about Adolescents and the Program (Principle 2)**

The training program should equip potential implementers with the necessary basic knowledge in these areas.

**Acquisition of Knowledge about the Curriculum Structure of the Program (Principle 3)**

To be an effective program implementer, the worker must know the program well. In particular, the sequencing details involved in different units of the curriculum should be made known to the potential program implementers.

**Cultivation of Appropriate Implementation Skills (Principle 4)**

To be effective program implementers, potential program implementers must possess appropriate teaching skills, such as communication techniques, varied teaching styles, active participation skills, and open discussion skills.

**Cultivation of Self-Reflection Skills (Principle 5)**

As positive youth development programs emphasize the importance of self-reflection of the program participants, potential program implementers should be helped to cultivate self-reflection skills regarding the implementation process and experiences of the program participants.

**Encouragement of Workers to Be Role Models (Principle 6)**

It is expected that program implementers of positive youth development programs have a strong influence on the program participants through their own experiences. As such, program implementers should be encouraged to serve as role models for the program participants to follow.

**Promotion of Motivation of the Trainees (Principle 7)**

As there are research findings showing that motivation of the program implementers is instrumental to program success, training programs should promote the willingness of potential program implementers to implement the program.

**Promotion of Self-Efficacy of the Trainees (Principle 8)**

As there are research findings showing that self-efficacy of the program implementers is instrumental to program success, training program participants should be helped to cultivate the belief that they possess the necessary competence to implement the program.

**Provision of Opportunities for Demonstration and Practice (Principle 9)**

To enable potential program implementers have actual personal experiences about the program to be delivered, demonstration with the participation of the trainees is important.

**Provision of Adequate Training Time (Principle 10)**

To enable potential program implementers to acquire adequate knowledge about adolescents and the designed program as well as to help them develop the qualities mentioned above, adequate training time should be provided for the training program.

**Consideration of Cultural Context in the Design of Training Program (Principle 11)**

Cultural adaptation and indigenization must be taken into account in developing the related program. Similarly, the related issues should be taken care of in the development of training program.

**Evaluation of Training Program (Principle 12)**

As good intention alone does not guarantee program effectiveness, evaluation of training programs via different evaluation methods would provide a triangulated picture about the effects of the training program.

In the Project P.A.T.H.S., evaluation of the training programs generally showed that the program participants were satisfied with the program, worker and effectiveness of the program [5, 6]. We believe that the positive training programs are instrumental to the success of the P.A.T.H.S. Project. In future, researchers should consider the following questions: what should be the optimal duration of training? Do the workers get any assistance from the school to release them to attend the training program? What should be the objectives of the training, such as focus on dissemination of knowledge on positive youth development programs or promoting positive attitude among the workers toward adolescents and positive youth development programs? As participants of the training programs may have different background, how can we ensure that the pitch and expectations in the training program are suitable for them?

### 2.3. Lesson  3: It is Important to Identify Determinants of Program Implementation Quality

In the Western literature, various factors governing the quality of program implementation have been identified. In the Project P.A.T.H.S., cross-case analyses revealed several conclusions [7]. First, several factors related to policy, people, program, process, and place (5 “P”s) were conducive to the successful implementation of the Tier 1 Program in the schools. Second, there were obstacles and difficulties with reference to 5 “P”s that impeded the quality of implementation. Third, policy support and people (especially commitment and passion of the principals, senior school administrators, and program implementers) factors are two main groups of factors influencing the quality of program implementation. Fourth, although there were different arrangements for program implementation, incorporation of the Tier 1 Program in the formal curriculum was a sound and viable strategy. Fifth, implementation of the Tier 1 Program in schools admitting students with high or low academic achievement was viable. Sixth, the program was generally perceived positively by the program participants and implementers. Finally, the program implementers perceived the program to be beneficial to the program participants.

In future, program developers should ask the following questions when they identify the possible process variables that may affect program implementation quality: how would principal support and interdisciplinary collaboration influence the quality of the implementation? What is the extent of program adherence and program fidelity in the implementation process? Is the morale of the program implementers high? If not, how can we promote the morale of the program implementers?

### 2.4. Lesson  4: The Need for Multiple Evaluation Strategies

For adolescent and positive youth development programs, one crucial issue is whether the programs can really benefit the program participants. In the Project P.A.T.H.S., the following principles are used to govern the evaluation of the project.

**Principle 1**

Objective changes in the participants via objective outcome indicators, including self-report rating scales are important areas considered in the evaluation.

**Principle 2**

The use of multiple objective outcome indicators would give a more complete picture about the program effects.

**Principle 3**

Participants' perceptions of the program (i.e., subjective outcomes) via client satisfaction survey are important data to be considered in the evaluation.

**Principle 4**

The views of the stakeholders, including those of the program developers, program implementers, administrators, and clients, are taken into account.

**Principle 5**

Program implementers' views (i.e., views of social workers and teachers) of the program are important data to be considered in the evaluation.

**Principle 6**

To adequately understand the program effects and the related contexts, qualitative data are collected.

**Principle 7**

Both quantitative and qualitative evaluation methods can give a more complete picture on the program effects.

**Principle 8**

Both process evaluation and outcome evaluation are included in the evaluation framework.

**Principle 9**

Triangulation of data collected from different sources by different methods would give a more in-depth picture of the program effects.

**Principle 10**

Validated measures are used to assess changes in the program participants.

**Principle 11**

Existing guidelines governing the evaluation of positive youth development or prevention programs of adolescent problems are observed.

**Principle 12**

Evaluation of the program is a continuous and ongoing process.

Based on the above principles, different evaluation strategies including objective outcome evaluation, subjective outcome evaluation based on both program participants and implementers, qualitative evaluation based on program implementers and participants, interim evaluation, process evaluation, and personal construct evaluation were adopted in the P.A.T.H.S. Project. In future, the following questions should be considered: what evaluation paradigm should be adopted to evaluate the program? In view of the existence of different strands and methods of qualitative evaluation methods, what qualitative evaluation methods can be used to assess the program? How can we triangulate evaluation findings based on different sources and different methods? How can we promote the workers' awareness of the importance of evaluation in the implementation of positive youth development programs?

### 2.5. Lesson  5: The Program Has Positive Impact for Different Stakeholders

There are several impacts of the project. First, the project has impact on secondary schools in Hong Kong regarding holistic youth development curriculum. It provides a useful and practical framework with roughly half of the secondary schools participating in the project. In addition, more than half of the participating schools have included the program in the formal curriculum. Second, evaluation based on multiple methods, multiple data, and multiple informants showed that different stakeholders of the project perceived that the project is beneficial to the program participants and the randomized group trial showed that students participating in the project showed better development than did the control participants. Third, the project is regarded as an important youth enhancement program by the government. It has been regarded as (a) an anti-poverty initiative by the Poverty Commission, (b) a key youth enhancement initiative by the Social Welfare Department, Government of the Hong Kong SAR, (c) a key adolescent prevention program (e.g., Panel on Child Fatality Review; Task Force on Youth Drug Abuse), and a program that can be used for antidrug education in schools (Resource Kit for Teachers on Anti-Drug Education). Fourth, the project has impact internationally. The project has been adapted and implemented in Shanghai and Macau. In addition, more than 100 international refereed publications have been generated from the project. The list of publications can be seen in the website of the project (http://www.paths.hk).

### 2.6. Lesson  6: Long-Term Sustainability of the Program Is Important

Given the positive impact of the project, the next question that should be seriously considered is how the program can be sustainable. In these few years, The Hong Kong Jockey Club Charities Trust has been very generous in supporting the project. What would happen if the financial support ceases in 2012? Basically, it is a matter of government policy on how positive youth development programs are treated in the school context. The related policy is important because there are numerous examples showing that home-baked programs are not working and positive youth development programs simply may not work [8]. Therefore, there is an urgent need for the government to rethink about how to promote programs on psychosocial competencies and how workable programs will be promoted in the school context. At the same time, attitude of the schools is also important. Schools should rethink the most important aspect of youth development and the role of positive youth development in holistic youth development.

 In the mean time, two initiatives are carried out to promote the long-term sustainability of the program. First, schools are encouraged to incorporate the program in the formal curriculum. Second, the Research Team will prepare e-learning and e-training packages so that workers can continue to use the packages after exhaustion of the funding. In future, several questions should be asked about long-term sustainability: is it conceptually and practically possible to incorporate the developed program in the regular curriculum or youth enhancement program? How can resources be redeployed to enhance the long-term sustainability of the program? How can the workers be conscious of the importance of updating the program materials?

### 2.7. Lesson  7: Conduct More Research on Positive Youth Development Programs in China

Despite the fact that the Project P.A.T.H.S. is very successful, it is noteworthy that more research in this area should be conducted. A review of the literature on prevention and positive youth development programs in Hong Kong showed that there were very few validated programs in the field, with the Project P.A.T.H.S. as a notable exception [9–11]. Of course, according to the hierarchy of evidence, further studies based on clinical trials carried out by one or several independent teams should be carried out. In the literature on adolescent prevention and positive youth development programs, despite progress in prevention science, effective programs in child and adolescent health are not widely adopted and difficulties in terms of translation of effective applied research to different real-life contexts and long-term sustainability exist. Ironically, even professionals working with young people such as pediatricians, nurses, social workers, and teachers do not have a good understanding of child and adolescent preventive programs. What are our responses to such a worrying picture?

Obviously, it is a long road to popularize child and adolescent prevention programs and widely adopt them in children and adolescent service contexts. Looking into the future, there are several areas of work that prevention scientists and positive youth development researchers should focus on. The first area is “*professional and public education*.” It is noteworthy that professionals working with children and adolescents are not familiar with prevention interventions. As such, training in public health, prevention science, and evidence-based practice should be included in the basic and continuing professional education programs for professionals working with youths. Training in terms of holistic adolescent development is also important. Besides prevention in physical health, psychosocial intervention targeting health and behavioral problems in adolescents should not be neglected in view of the rising prevalence of related behavior in different contexts. It is obvious that the focus of the public and professionals is put more on the prevention of physical illness than psychosocial and behavioral problems in children and adolescents. Obviously, it is necessary to have a paradigm shift in understanding the importance of prevention in different domains of children and adolescents. Besides professional education, public education is also important. While the general public commonly has high expectation about prevention work for physical illness (e.g., vaccines), expectation about the quality of psychosocial preventive initiatives (e.g., preventive drug education program) is not high. While the notion of primary prevention is endorsed by the general public, it seems that it is primarily confined to physical illnesses than psychosocial problems.

The second direction relates to the “*youth vision*” of the government. Government officials must appreciate the importance of primary prevention programs in children and adolescents which will eventually reduce health spending and social costs. Furthermore, without policy support, implementation of children and adolescent prevention programs, particularly school-based programs, would not be possible. As political processes and cultural ideologies strongly affect the policies, development, and implementation of adolescent prevention programs, more attention should be paid to politics and the “political will” involved in advocating for youth prevention programs.

Finally, “*dissemination of knowledge about effective programs*” is another direction to work on. Dissemination of research on effective programs for professionals working with children and adolescents is important to engage professionals to use the developed programs. Similarly, strengthening the communication between the government and prevention scientists is a key to the development of prevention-focused policies. Furthermore, educating the public (particularly parents and adolescents themselves) about the value of prevention will promote the use of such programs. In the long run, credible databases documenting exemplary and promising programs are also important. It is noteworthy that there are no such credible databases in different Chinese communities, including Hong Kong.

When we take the first letter in these three areas (“professional and public education”, “youth vision,” and “dissemination”), the acronym is “pyd” (i.e., positive youth development). Definitely, joining hands with the government, other allied professionals, parents, adolescents, and the general public, prevention and positive youth development scientists have to work out strategies that can promote children and adolescent prevention work at the national, regional, and global contexts, particularly in different Chinese communities.
